# Methylene Blue Delivery Mediated by Focused Ultrasound-Induced Blood–Brain Barrier Disruption Reduces Neural Damage and Amyloid-Beta Plaques by AQP-4 Upregulation

**DOI:** 10.3390/biomedicines10123191

**Published:** 2022-12-08

**Authors:** Hyo Jin Choi, Mun Han, Byeongjin Jung, Yu-Ri Hong, Seulgi Shin, Sungsu Lim, Eun-Hee Lee, Yun Kyung Kim, Juyoung Park

**Affiliations:** 1 Medical Device Development Center, Daegu-Gyeongbuk Medical Innovation Foundation (K-MEDI Hub), 80 Cheombok-ro, Dong-gu, Daegu 41061, Republic of Korea; 2 Daegu Technopark, 46-17, Seongseogongdan-ro, Dalseo-gu, Daegu 42716, Republic of Korea; 3 Biomedical Research Institute, Joint Institute for Regenerative Medicine, Kyungpook National University Hospital, Daegu 41940, Republic of Korea; 4 Center for Brain Disorders, Brain Science Institute, Korea Institute of Science and Technology (KIST), Seoul 02792, Republic of Korea; 5 College of Future Industry, Department of High-Tech Medical Device, Gachon University, 1342, Seongnam-daero, Sujeong-gu, Seongnam-si 13120, Republic of Korea

**Keywords:** blood–brain barrier, focused ultrasound, methylene blue, Alzheimer’s disease, aquaporin-4

## Abstract

Alzheimer’s disease (AD) is the most prevalent neurodegenerative disease worldwide, causing progressive cognitive decline, memory impairment, and neurological deficits. Methylene blue (MB), an antioxidant, has emerged as a potential drug for the treatment of AD owing to its cognitive improvement and neuroprotective functions. Despite the small molecular size of MB, which can cross the BBB, the therapeutic effective dosage using a BBB-permeable delivery system in a specific brain localization remains unclear. In this study, we presented magnetic resonance–guided focused ultrasound (MRgFUS) as a delivery system to enhance BBB permeability for the effective treatment of AD. MRgFUS using two ultrasound intensities (0.25 and 0.32 MPa) was used to intravenously deliver MB to the hippocampal region. Compared with treatment with 0.25 MPa FUS, treatment with 0.32 MPa FUS significantly enhanced MB brain accumulation. Deposition of amyloid-β (Aβ) plaques and neural cell damage was significantly reduced in 0.32 MPa FUS/MB-treated APP/PS1 mice. Furthermore, aquaporin-4 expression increased significantly in the 0.32 MPa FUS and 0.32 MPa FUS/MB groups without glial fibrillary acidic protein activation. The results from this study demonstrate that FUS improved MB delivery to the brain, and FUS/MB combination treatment reduced the number of Aβ plaques. This study revealed the potential of FUS-BBBD as an effective strategy to enhance the efficacy of therapeutic drugs for AD.

## 1. Introduction

Alzheimer’s disease (AD), a neurodegenerative disorder, is the most common form of senile dementia [1]. Although the pathogenic mechanism of AD remains unclear, the pathological hallmarks of AD include the extracellular deposition of amyloid-β (Aβ) plaques and intracellular neurofibrillary tangles [2]. The progressive accumulation of Aβ oligomers is theorized to be caused by an imbalance between Aβ production and clearance, which leads to the initiation of AD pathology [3,4]. There is increasing evidence to support the theory that in an age-related decrease, paravascular recirculation clearance is responsible for the accumulation of Aβ plaques in the brain parenchyma [5,6]. Therefore, Aβ oligomers, which are neurotoxic aggregates, are active targets for AD treatment. Several therapeutic options, such as monoclonal antibodies, inhibitors, and proteases, which can reduce the accumulation of Aβ oligomers are currently under development or being assessed in clinical trials [7,8]. However, the limited ability of these therapeutic drugs to reach the central nervous system (CNS) poses a challenge to effective AD treatment. One of the major hurdles to the delivery of drugs to the CNS is the blood–brain barrier (BBB), which is a physical and functional barrier comprising endothelial cells, astrocytes, and pericytes, which hinders drug delivery to the brain [9].

Methylene blue (MB), a traditional mitochondria-targeting antioxidant, is clinically used to treat methemoglobinemia, malaria, cyanide poisoning, and carbon monoxide poisoning. In recent years, MB has emerged as a potent CNS-targeting drug owing to its ability to cross the BBB and its beneficial effects on memory enhancement and neuroprotection [10,11]. Owing to its metabolic-enhancing and antioxidant properties, the efficacy of MB in AD [12], Parkinson’s disease (PD) [11], amyotrophic lateral sclerosis [13], traumatic brain injury [14], and ischemic stroke has been investigated [15]. Paban et al. found that MB treatment protected the brains of transgenic mice against cognitive impairment and concomitantly reduced brain amyloid load and mitochondrial dysfunction [12]. In addition, MB significantly reduced infarct volumes 24 h post-cerebral ischemia–reperfusion injury [15]. Unlike most CNS-targeting drugs, although small molecules such as MB (molecular size ≤ 400 Da) can cross the BBB, high doses of these drugs are necessary to achieve therapeutic effects [16]. Therefore, there is a need for the development of an efficient BBB-permeable delivery system that enables the systemic administration of MB at a low but clinically effective dose. 

Focused ultrasound (FUS) in combination with intravenous microbubbles is an emerging non-invasive drug delivery system that opens the BBB, thus facilitating drug delivery to the CNS [17,18]. The use of FUS for drug delivery has several advantages, including targeted delivery using magnetic resonance imaging (MRI) guidance [17] and drug delivery irrespective of the molecular size (small compounds to cells) [19,20,21]. In addition, well-defined FUS assures a safe, reproducible, and transient BBB opening [22,23,24]. Focused-ultrasound-induced BBB disruption (FUS-BBBD) in combination with therapeutic drugs has been used in preclinical disease models, such as brain tumors [25,26], PD [27,28], and AD [29,30]. Earlier studies reported that anti-Aβ and anti-tau antibodies delivered using FUS significantly reduced Aβ plaque number and enhanced memory/cognitive improvement in a transgenic mouse model of AD [29,31,32]. In addition, FUS-BBBD reduced plaque burden, triggered neuronal plasticity, and enhanced spatial memory with a single treatment [33,34]. More recently, several studies have hypothesized that FUS-BBBD may enhance the glymphatic pathway (CNS clearance system) via modulation of water dynamics in healthy [35] and AD-like animals [36]. 

The glymphatic system is a brain-wide drainage system that facilitates the clearance of interstitial metabolic waste products from the brain parenchyma [5,37]. It is an integral part of the cerebrospinal fluid (CSF)–interstitial fluid (ISF) exchange in the brain that is driven by the convective flow of CSF, which moves solutes from the parenchyma via the perivascular spaces [38]. Recent studies reveal that water dynamics in the perivascular spaces are closely associated with the aquaporin-4 (AQP-4) function [39,40,41]. AQP4 is the most abundant water channel protein in the brain, particularly predominant in the perivascular astrocyte end-feet. This protein contributes to ionic and osmotic homeostasis by facilitating the bidirectional flow of water and small uncharged solutes [42]. Recent accumulating evidence suggests that AQP-4 connects the astrocyte intracellular space and perivascular extracellular space, thereby facilitating the convective flow of CSF into the interstitial space, essential for the glymphatic pathway [5,42]. Earlier studies showed that AQP-4-deficient mice exhibited slow CSF influx and reduced solute clearance in the brain interstitium, supporting the hypothesis that the glymphatic system is AQP-4-dependent [5,43]. Our earlier study was the first to report that FUS-BBBD could induce local water diffusion via increased AQP-4 expression in healthy rats [35]. However, the effect of FUS-BBBD to induce AQP-4 expression in AD models remains unclear.

In this study, we demonstrated that FUS-BBBD-mediated MB delivery to the CNS effectively reduced Aβ plaque deposition and neural cell damage in APP/PS1 transgenic mouse brains. Furthermore, AQP-4 expression was significantly higher in the FUS-treated region, suggesting the possibility of waste removal via glymphatic system activation. This study suggests the therapeutic potential of FUS in combination with MB for AD treatment and provides novel insights into the molecular mechanisms underlying AD.

## 2. Materials and Methods

### 2.1. Animal Handling and Experimental Design

All animal experiments were approved and conducted in accordance with the guidelines of the Institutional Animal Care and Use Committee of Daegu-Gyeongbuk Medical Innovation Foundation (IACUC No. 17110201-01). APP/PS1 transgenic mice (12–14-month-old, 30–40 g) were obtained from the Korea Institute of Science and Technology (Seoul, Republic of Korea). Institute of Cancer Research (ICR) mice (5 weeks old, 25–30 g) were purchased from Orient Bio Inc. (Sungnam, Republic of Korea). The animals were exposed to a 12 h light/dark cycle at a controlled temperature (23 °C ± 2 °C) and humidity conditions (40–45%) with free access to food and water. To evaluate the effect of FUS-BBBD with and without MB, the mice were randomized and assigned to four groups according to the experimental design described in Table 1. In the 0.25 pressure amplitude (MPa)-treated group, the animals were divided into the following two sub-groups: (1) FUS without MB (denoted as vehicle, N = 5) and (2) FUS in combination with MB (denoted as MB, N = 6). In the 0.32 MPa-treated group, the animals were divided into the following two sub-groups: (1) FUS without MB (denoted as vehicle, N = 6) and (2) FUS in combination with MB (denoted as MB, N = 6).

### 2.2. MRI-Guided FUS (MRgFUS) System

**Figure 1:**
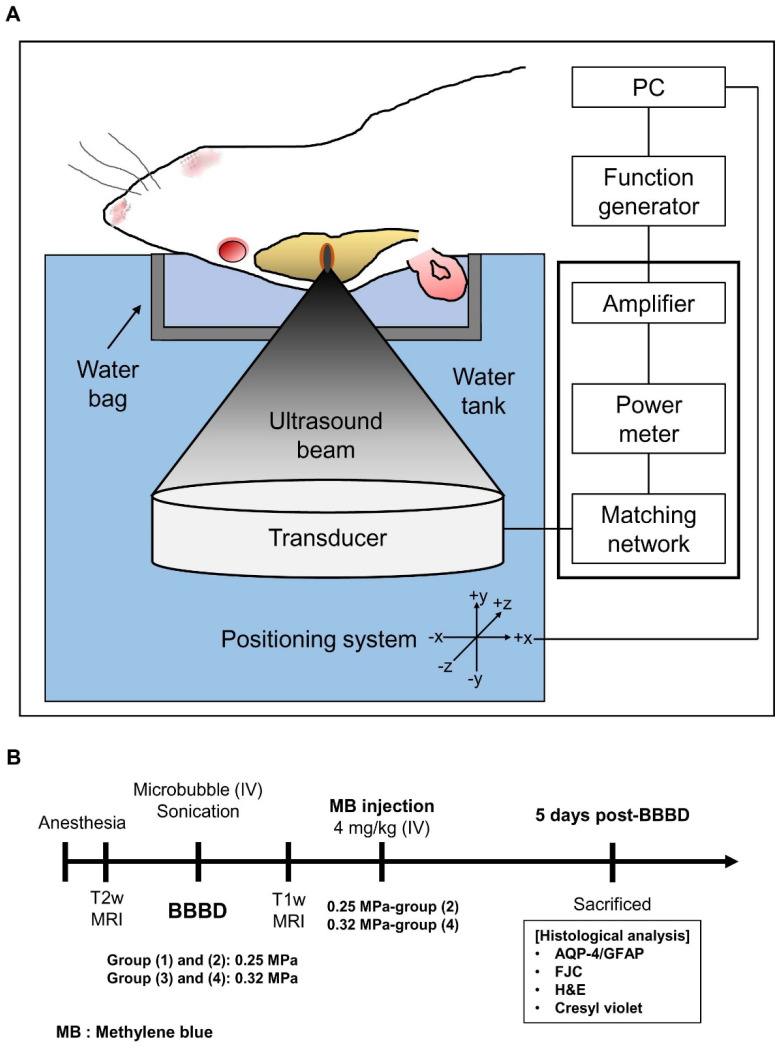
Schematic representation of the MRgFUS system and experimental procedures. (**A**) The mouse is placed in a supine position on the plastic bed with its head submerged in a water bag. The focal-targeted region was exposed to the upward ultrasound wave using MRI and a PC-controlled positioning system. (**B**) Animals were first exposed to MRgFUS directed to the hippocampal region of the right hemisphere. After sonication, MB (4 mg/kg body weight) was intravenously injected into the 0.25 MPa (2) and 0.32 MPa (4) groups. Animals were sacrificed 5 days post-BBBD and histological assessments were conducted.

The MRgFUS system (RK-100; FUS Instruments, Toronto, Canada) was used for BBB disruption (BBBD), as described in an earlier study [44]. A schematic representation of the MRgFUS system is shown in Figure 1A. A spherical piezoelectric FUS transducer (diameter: 75 mm, radius of curvature: 60 mm, center frequency: 1.1 MHz; FUS Instruments) was used to generate concentrated ultrasound. The width and length of the 3 dB focal region of the transducer were 1.5 and 6 mm, respectively. An arbitrary waveform generator (33220A; Agilent, Santa Clara, CA, USA) and a radio-frequency power amplifier (4010 L; E&I, Rochester, NY, USA) were used to drive the transducer signals. The transducer was submerged in a tank filled with degassed water. 

A clinical 3T MRI system (MAGNETOM Skyra; Siemens Healthineers, Erlangen, Germany) and preclinical 9.4T MRI system (BioSpec 94/20 USR; Bruker, Billerica, MA, USA) were used for anatomical mapping of target foci in the FUS system. Magnetic resonance (MR) images were fed into to the MRgFUS system, and the coordinates of the two systems were synchronized. T1-weighted (T1w) MR images were used to evaluate BBBD, and T2-weighted (T2w) MR images were used to select the target focal region. The sequences used and imaging parameters are summarized in Table 2.

### 2.3. Procedure for BBBD 

The MRgFUS system for BBBD has been described previously [44]. The animals were anesthetized intramuscularly using a mixture of Zoletil (25 mg/kg body weight; Virbac Laboratories, Carros, France) and Rompun (4.6 mg/kg body weight; Bayer, Leverkusen, Germany). The mouse was secured in a supine position on an MR-compatible bed after inserting an angiocatheter into the tail vein. The target region for BBBD was the hippocampus of the right hemisphere, and target coordinates were obtained using the T2w MR images before sonication. Sonication was started 10 s after 1:50 diluted Definity^®^ (Lantheus Medical Imaging, Inc., North Billerica, MA, USA) was infused using an automated syringe pump (Harvard Apparatus, Holliston, MA, USA). Ultrasound (acoustic pressure = 0.25 or 0.32 MPa depending on the experimental group) was delivered at a 10 ms tone burst with a pulse repetition frequency of 1 Hz for 120 s. Post-FUS exposure, MB (4 mg/kg body weight; Sigma-Aldrich, St. Louis, MO, USA) was immediately administered to the animals in the 0.25 and 0.32 MPa groups via bolus injection. T1w MR images obtained after the administration of gadolinium-tetraazacyclododecane-tetraacetic acid (Gd-DOTA) (Dotarem^®^, Guerbet, Roissy CdG Cedex, France) to all groups were used to confirm BBBD. The mice were sacrificed 4 h or 5 days post-sonication for further experimentation (Figure 1B). 

### 2.4. MR Image Analysis

All temporal variations in contrast-enhanced T1w MR images (at 5, 10, and 15 min) were normalized using the contralateral normal region and subtracted from the preinjection T1w MR images (at 0 min). The region of interest (ROI) is represented by a circle (4-pixel radius) corresponding to the 1.5 mm focal area of the transducer (2.56 pixels/mm). The relative signal intensity (*R_t_*) was calculated using the following equation:

$$R_{t}\left(\%\right)=\frac{\left(T1_{t}-T1_{pre}\right)}{T1_{pre}}\times 100$$

In the above equation, *T*1*_pre_* indicates the average intensity of T1w MR images of the ROI before the preinjection of contrast agents, while *T*1*_t_* indicates the average intensity of T1w MR images of the ROI post-injection of the contrast agents into the sonication target region at 5, 10, and 15 min.

### 2.5. In Vivo Imaging System (IVIS)

ICR mice (N = 10) were used to measure the brain accumulation of FUS-BBBD-delivered MB using the IVIS spectrum CT (PerkinElmer, Waltham, MA, USA). The mice were exposed to acoustic pressure of different intensities (0.25 and 0.32 MPa), and preinjection brain fluorescence images (0 min) using in vivo imaging were captured. Subsequently, MB was intravenously administered (4 mg/kg body weight), and fluorescence images were captured at 1, 5, 30, 60, and 240 min. Mice in the control group were injected with the same dose of MB but without FUS treatment. MB fluorescence was detected using 670 nm excitation and 690 nm emission filters. The images were evaluated using Living Image software version 4.7.4 (PerkinElmer).

### 2.6. Quantification of Aβ Plaque Deposition

To assess Aβ plaque deposition, brain tissues were stained with thioflavin-S (Sigma-Aldrich, St. Louis, MO, USA). The mice were sacrificed 5 days post-sonication and transcardially perfused with 0.9% saline. The brain tissue was post-fixed with 4% paraformaldehyde at 4 °C for 3 days. The tissue was cut into 30 μm-thick slices using a vibrating blade microtome (VT1200S; Leica, Wetzlar, Germany). Free-floating tissues were placed in 1% thioflavin-S aqueous solution for 5 min. The tissues were immersed in Hoechst33342 (10 μg/mL dissolved in distilled water) for 5 min to counterstain the nuclei. After washing with phosphate-buffered saline, tissue slices were mounted on saline-coated slides and cover-slipped using a fluorescent mounting medium (Dako, Carpinteria, CA, USA). 

### 2.7. Immunofluorescence

Briefly, 30 μm sections of the formalin-fixed brain were sectioned on a vibratome and collected in PBS containing 0.01% sodium azide (Sigma-Aldrich). Free-floating tissues were treated for antigen retrieval (Abcam, Cambridge, UK) in the microwave by heating for 20 min. The tissues were permeabilized in 0.25% Triton X-100 for 1 h, followed by blocking with 10% normal goat serum for 1 h at room temperature. Subsequently, tissue sections were incubated with antibodies against the glial fibrillary acidic protein (GFAP, 1:400, Sigma-Aldrich) and aquaporin-4 (AQP-4, 1:500, Abcam) overnight at 4 °C. The tissues were incubated with Alexa Fluor 488-labeled goat anti-mouse-IgG (1:1000; #A32723, Invitrogen, Carlsbad, CA, USA) or Alexa Fluor 633-labeled goat anti-rabbit-IgG (1:1000; #A32731, Invitrogen), correspondingly, for 1 h at room temperature. The slides were mounted with fluorescence mounting medium (Dako).

### 2.8. Fluoro-Jade C (FJC) Staining

A commercial ready-to-dilute FJC staining kit (Biosensis Inc., Tebarton, SA, Australia) was used to detect degenerating neurons, as described previously [35]. Free-floating tissue slices were mounted onto saline-coated glass slides. The tissue slides were incubated with a 10% (*v*/*v*) potassium permanganate solution for 15 min and subsequently rinsed using distilled water. The slides were transferred to a mixture of 20% FJC and 20% (*v*/*v*) 4,6-diamidino-2-phenylindole (DAPI), which was dissolved in 0.1% acetic acid. The slides were dried and cover-slipped using dibutyl phthalate polystyrene xylene mounting medium (Sigma-Aldrich).

### 2.9. Histopathology

For histopathological staining, the brain tissue samples were embedded in paraffin and cut serially into 4 μm-thick axial sections using a microtome (HistoCore AUTOCUT; Leica, Wetzlar, Germany). Hematoxylin and eosin (H&E) and cresyl violet staining were conducted to identify tissue/neuronal damage post-sonication. Paraffin-embedded sections were deparaffinized and rehydrated prior to staining. Brain tissue slides were stained using an H&E staining kit (Vector Laboratories, Burlingame, CA, USA) or 0.1% cresyl violet acetate solution (Sigma-Aldrich).

### 2.10. Image Analysis

All images were captured at 20× magnification with a digital slide scanner (Axio Scan.Z1; Carl Zeiss, Oberkochen, Germany) and analyzed using ZEN3.4 light software (Carl Zeiss) for fluorescence intensity quantification and count. Regions of interest (ROIs) were located within the ultrasound-treated region corresponding to the contrast-enhancing regions of axial T1w MR images. Within each ROI (1.32 mm by 0.72 mm), two regions were assessed, one in the cortex and one in the hippocampus. Fluorescence intensity was defined as the relative change in fluorescence in the ROIs compared with that in the contralateral regions not exposed to ultrasound.

### 2.11. Statistical Analysis

All values are expressed as mean ± standard error of the mean (SEM). The statistical significance of differences between the control and experimental groups was determined using a two-tailed unpaired *t*-test and two-way analysis of variance (ANOVA) with Tukey’s test using GraphPad Prism 8 (GraphPad Software, Inc., San Diego, CA, USA). Statistical significance was set at *p* < 0.05.

## 3. Results

### 3.1. Confirmation of MRgFUS-Induced BBBD

**Figure 2:**
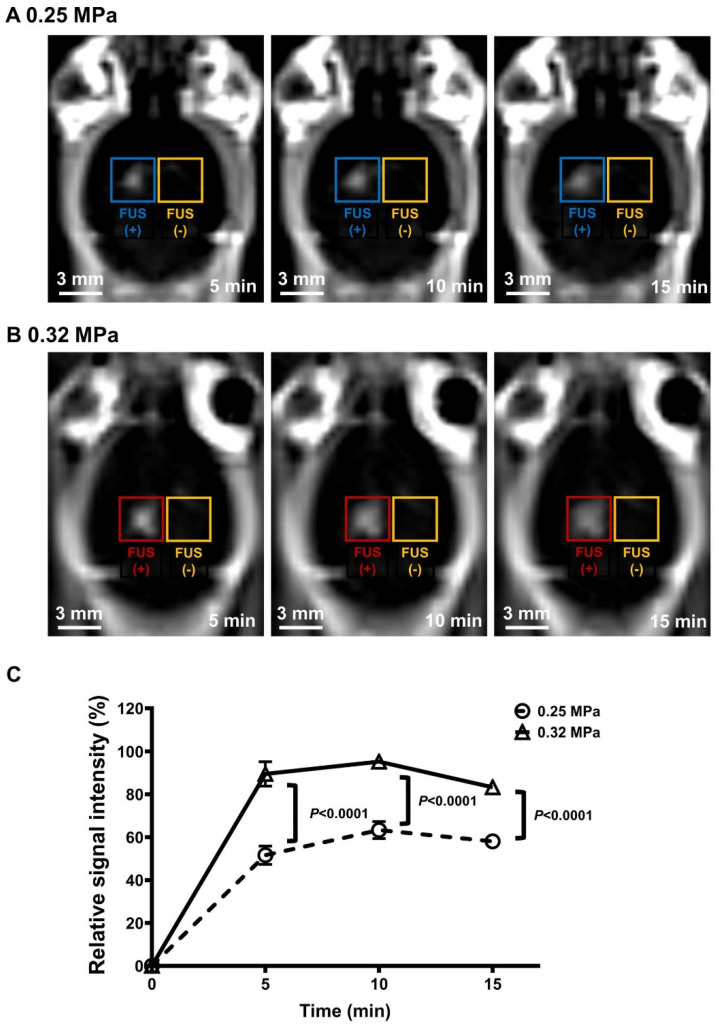
Confirmation of BBBD using two different FUS intensities. (**A**,**B**) Representative T1w MR images captured from the 0.25 MPa (**A**) and 0.32 MPa (**B**) groups at different time points (5, 10, and 15 min). (**C**) The enhancement of gadolinium signal intensity was calculated by subtracting the signal intensity in the FUS-treated region (0.25 MPa FUS: blue box, circle, and dashed line; 0.32 MPa FUS: red box, triangle, and solid line) from the background intensity in the contralateral region (no-FUS: yellow box). Data are presented as mean ± SEM (N = 4 in each group). Statistical significance was analyzed using a two-tailed unpaired *t*-test. Scale bars represent 3 mm in (**A**,**B**).

Two acoustic pressure intensities were utilized in this study to evaluate the synergistic effect of FUS-BBBD in combination with MB. The extent of MRgFUS-induced BBBD was confirmed based on the analysis of contrast-enhanced T1w MR images. MR images were captured 5, 10, and 15 min post-gadolinium-diethylenetriaminepentaacetic acid (Gd-DTPA) injection, and representative images are shown in Figure 2A,B. The relative signal intensity in the BBBD region was increased to a mean intensity of 57.7 ± 5.8% in the 0.25 MPa-treated group and 89.3 ± 6.0% in the 0.32 MPa-treated group, compared with the signal intensity in the contralateral region (Figure 2C). The difference in the BBB permeability between the 0.25 MPa-treated and 0.32 MPa-treated groups post-sonication was found to be statistically significant (*p* < 0.0001) and experimentally feasible.

### 3.2. FUS-BBBD-Mediated Enhancement of MB Delivery to the CNS

**Figure 3:**
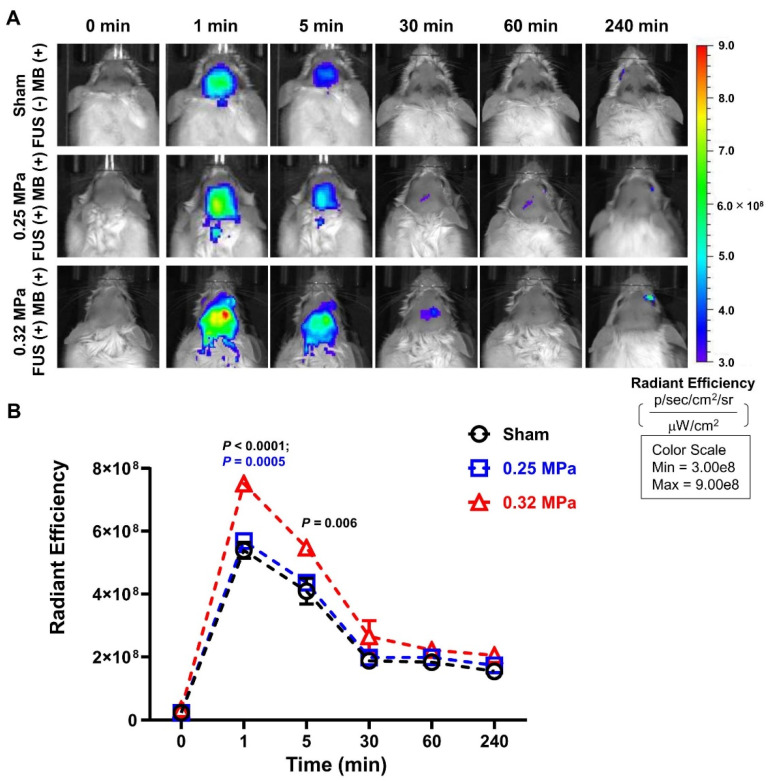
Detection of MB delivered to the brain using the IVIS. (**A**) Representative images of in vivo fluorescence emission were captured at fixed time points (0, 1, 5, 30, 60, and 240 min). (**B**) Radiant efficiency of fluorescence emitted from the brain post-MB injection was quantified using the IVIS (sham group: black circle; 0.25 MPa group: blue square; 0.32 MPa group: red triangle). Data are presented as mean ± SEM (N = 4 for the sham group, N = 3 for the 0.25 MPa, and N = 3 for 0.32 MPa groups). Statistical significance was analyzed using two-way ANOVA with Tukey’s multiple-comparison test.

The near-infrared fluorescence signal of MB was monitored in a time-dependent manner to quantitatively evaluate FUS-BBBD-mediated MB delivery across the BBB. As shown in Figure 3A, the radiant efficiency of MB from the brain was found to be 5.4 × 10^8^ in the sham group (FUS (−) MB (+)), indicating low BBB permeability. One-minute post-FUS treatment, the average radiant efficiency increased to 5.9 × 10^8^ and 7.5 × 10^8^ (*p* < 0.0001) in the 0.25 MPa-treated and 0.32 MPa-treated groups, respectively (Figure 3B). The fluorescence intensity in the 0.25 MPa-treated group was slightly higher than that in the control group, but there were no significant differences. In contrast, 5 min post-injection the 0.32 MPa-treated group exhibited an average MB radiant efficiency of 5.5 × 10^8^, which was 1.3 times significantly higher (*p* = 0.006) than the radiant efficiency observed in the control group (4.1 × 10^8^) (Figure 3B). While MB delivered without the use of FUS was rapidly reduced within 30 min, the MB delivered via FUS-BBBD in the 0.32 MPa-treated group was retained for up to 30 min (Figure 3A,B). These results suggest efficient FUS-BBBD-mediated MB delivery and retention in the 0.32 MPa-treated group compared with the sham and 0.25 MPa-treated groups.

### 3.3. FUS-BBBD-Mediated MB Drug Delivery Decreased the Number of Aβ Plaques

**Figure 4:**
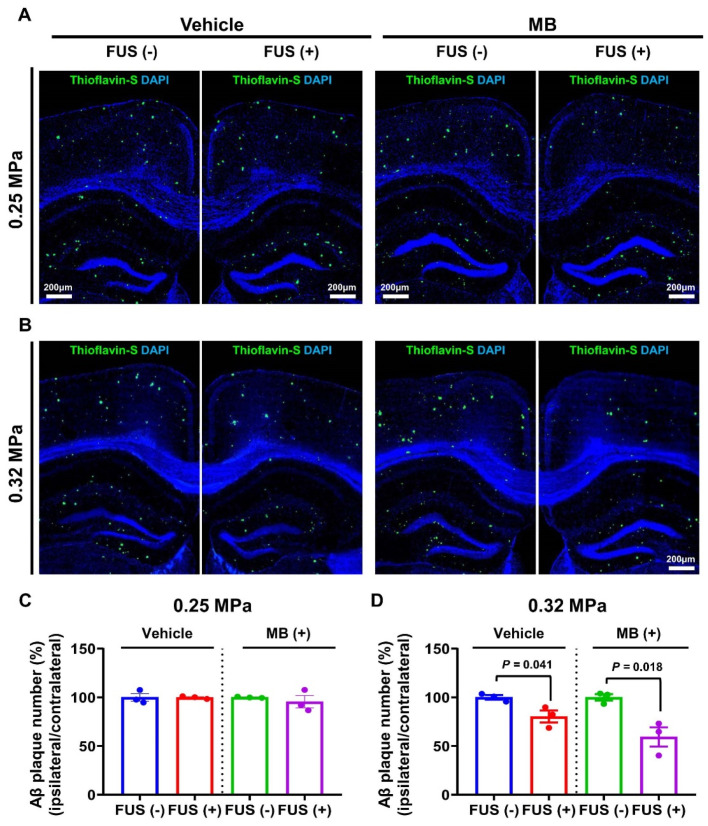
Aβ plaque deposition in FUS-treated and FUS/MB-treated APP/PS1 mice. (**A**,**B**) Thioflavin-S staining in the cortex and hippocampal regions revealed representative Aβ plaque deposition (green fluorescence) in the 0.25 MPa (**A**) and 0.32 MPa (**B**) groups. (**C**,**D**) The number of Aβ plaques in the cortex and hippocampal regions of animals in the 0.25 MPa (**C**) and 0.32 MPa (**D**) groups was determined. Data are presented as mean ± SEM (number of animals, N = 3; number of slices of each specimen, n = 3). Statistical significance was analyzed using a two-tailed unpaired *t*-test. Scale bars represent 200 μm in (**A**,**B**).

The therapeutic effects of FUS-BBBD-mediated MB drug delivery were evaluated by analyzing plaque pathology in the BBBD region (cortex and hippocampus in the right hemisphere) in comparison with the pathology in the contralateral region (cortex and hippocampus in the right hemisphere). To verify the therapeutic effects, mice were assigned to four treatment groups. The four groups listed in Table 1 are as follows: (1) 0.25 MPa FUS (single FUS in the right hippocampus), (2) 0.25 MPa FUS/MB (treated with FUS and 4 mg/kg body weight intravenous (IV) dose of MB), (3) 0.32 MPa FUS (single FUS in the right hippocampus), and (4) 0.32 MPa FUS/MB (treated with FUS and 4 mg/kg body weight IV dose of MB). The number of Aβ plaques in the cortex and hippocampus of the APP/PS1 mice was evaluated after 5 days (Figure 4). No significant change in the number of Aβ plaques was observed in both 0.25 MPa-treated groups (single FUS and FUS/MB-treated groups) (Figure 4A,C). However, the number of Aβ plaques in the BBBD region was significantly reduced to 19.7 ± 10.7% (*p* = 0.041) in the 0.32 MPa FUS group and 40.6 ± 17.1% (*p* = 0.018) in the 0.32 MPa FUS/MB group in comparison with the number of plaques in the contralateral region (Figure 4B,D). These results indicate that 0.32 MPa FUS and 0.32 MPa FUS/MB decreased the Aβ plaque number. Among these two groups, 0.32 MPa FUS/MB elicited the most significant plaque-reducing effect.

### 3.4. Changes in AQP-4 Expression

**Figure 5:**
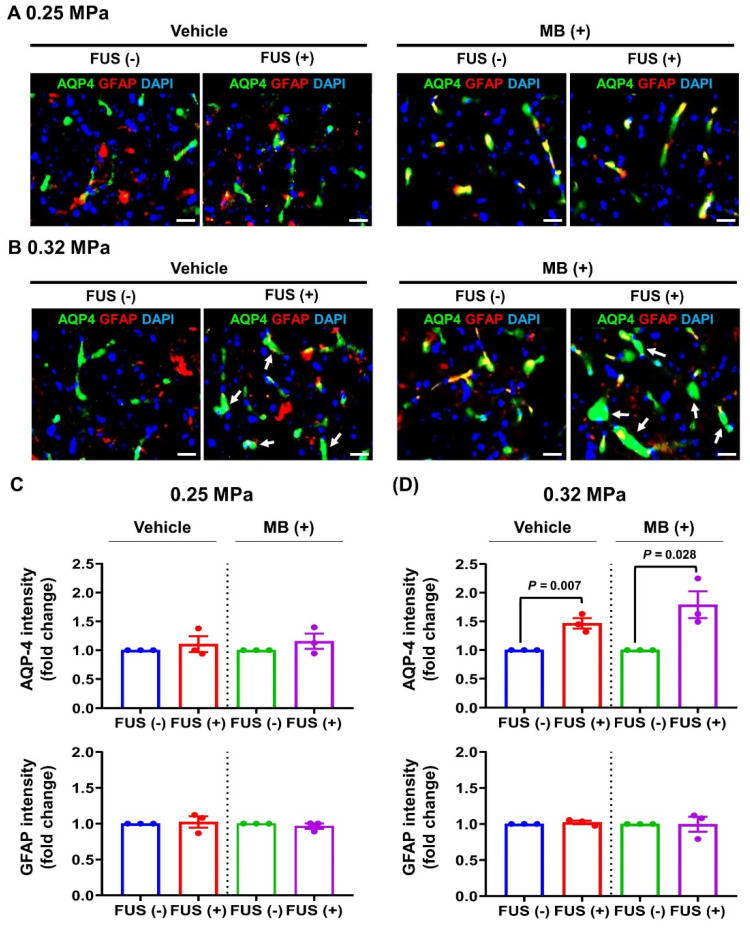
Changes in astrocytic AQP-4 expression in FUS-treated and FUS/MB-treated APP/PS1 mice. (**A**,**B**) Representative immunofluorescence images showing AQP-4 (green, a water channel protein) and GFAP (red, an astrocyte marker) intensities in the 0.25 MPa (**A**) and 0.32 MPa (**B**) groups. White arrows indicate upregulated AQP-4 expression (green). (**C**,**D**) The relative fluorescence intensity of AQP-4 (**C** and **D**, upper) and GFAP (**C** and **D**, bottom) was measured from 6 ROIs per hemisphere and normalized using the relative fluorescence intensity values from the non-FUS region. Data in the bar graph are presented as mean ± SEM (N = 3). Statistical significance was analyzed using a two-tailed unpaired *t*-test. Scale bars represent 20 μm in (**A**,**B**).

Based on earlier evidence that FUS-BBBD induced AQP-4 expression in the target region [35], we evaluated if FUS-BBBD could induce AQP-4 expression in APP/PS1 mice. AQP-4 expression and astrocyte activation in the 0.25 MPa FUS and 0.25 MPa FUS/MB groups 5 days post-BBBD remained unchanged (Figure 5A,C). In contrast, AQP-4 expression was significantly increased in the 0.32 MPa-treated groups 5 days post-BBBD (Figure 5B). The signal intensity of AQP-4 expression was 1.47 ± 0.09-fold higher in the 0.32 MPa FUS group than the signal intensity in the contralateral hemisphere. AQP-4 expression in the 0.32 MPa FUS/MB group was 1.73 ± 0.26-fold higher than AQP-4 expression in the non-FUS hemisphere (Figure 5D). Astrocyte activation that usually accompanies increased AQP-4 expression was not observed in the 0.32 MPa FUS and 0.32 MPa FUS/MB groups 5 days post-BBBD (Figure 5B,D). These results suggest that the FUS-BBBD induced by exposure to 0.32 MPa in combination with an anti-oxidative drug (MB) induced AQP-4 expression without astrocyte activation in AD mice. 

### 3.5. Effect of MRgFUS-Mediated MB Delivery on Neural Cell Damage

**Figure 6:**
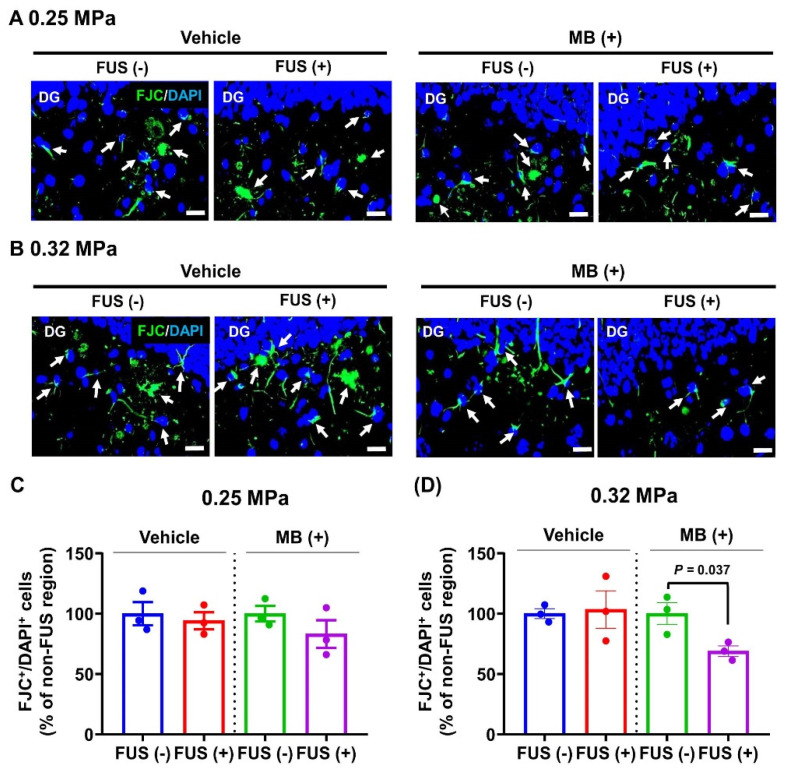
Evaluation of neural cell damage in FUS-treated and FUS/MB-treated APP/PS1 mice. (**A**,**B**) Representative photomicrographs of FJC (damaged neural cell marker) staining (green) captured from the DG of the hippocampal region of animals in the 0.25 MPa (**A**) and 0.32 MPa (**B**) groups. The number of FJC-positive cells in the cortex and hippocampal regions in accordance with the FUS target (ROI = 5.1 mm^2^ in each hemisphere) was determined. White arrows indicate double-positive cells for FJC and DAPI (a nucleus marker) (green). (**C**,**D**) The number of FJC/DAPI-double-positive cells in the cortex and hippocampal regions of animals in the 0.25 MPa (**C**) and 0.32 MPa (**D**) groups was determined. Data in the bar graph are presented as mean ± SEM (N = 3). Statistical significance was analyzed using two-tailed unpaired *t*-test. Scale bars represent 20 μm in (**A**,**B**).

To determine the effect of FUS-BBBD and FUS-BBBD-mediated MB delivery on neural cell damage, FJC staining was performed in the four treatment groups (Figure 6). The number of FJC-positive cells in the BBBD region (cortex and hippocampus) post-sonication was counted to evaluate neural cell damage (Figure 6A,B). Upon exposure to 0.25 MPa, there was no significant difference in the number of FJC-positive cells in the BBBD region compared with their number in the contralateral hemisphere (0.25 MPa FUS, 529 ± 68.5 cells/field (BBBD region), and 561.7 ± 93.6 cells/field (contralateral hemisphere); 0.25 MPa FUS/MB, 398.3 ± 95.2 cells/field (BBBD region), and 479.3 ± 53.7 cells/field (contralateral hemisphere); Figure 6C). Furthermore, no significant difference in the number of FJC-positive cells was observed in the 0.32 MPa FUS group compared with their number in the contralateral hemisphere (616 ± 159.7 cells/field (BBBD region) and 595.7 ± 42.1 cells/field (contralateral hemisphere); Figure 6D). However, the number of FJC-positive cells in the 0.32 MPa FUS/MB group was significantly lower compared with their number in the contralateral region (31.1% reduction; 561.3 ± 88.2 (contralateral region) and 387 ± 42 (FUS/MB); *p* = 0.037; Figure 6B,D). These results suggest that FUS-BBBD-mediated MB delivery reduced neural cell damage in the brain region affected by AD pathology. 

### 3.6. Histopathological Examination

**Figure 7:**
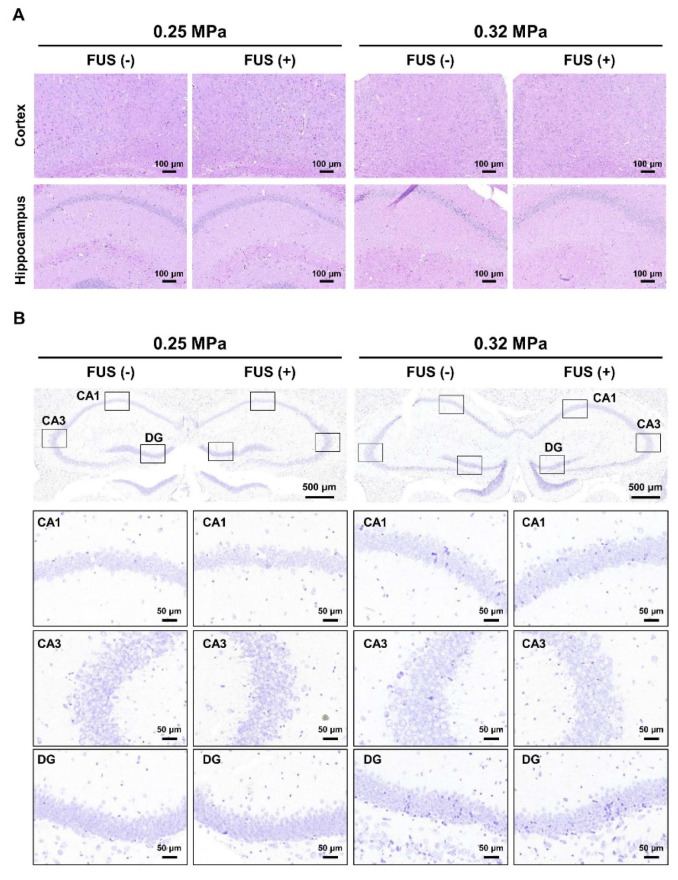
Histopathological evaluation of the brain tissue of APP/PS1 mice subjected to FUS-BBBD. (**A**) Representative H&E-stained images showed the cortex (top) and hippocampal (bottom) regions after 0.25 MPa FUS (two columns on the left) and 0.32 MPa FUS (two columns on the right). Scale bars represent 100 μm (**B**) Representative images of the CA1, CA3, and DG hippocampal regions stained using cresyl violet 5 days post-BBBD. Scale bars represent 500 μm for images of the hippocampus region (**B**, upper) and 50 μm for enlarged images (**B**, bottom).

To determine the effect of two different sonication intensities on the occurrence of microbleeding in APP/PS1 mice, we performed histopathological analysis of the tissues subjected to unilateral sonication of the hippocampal regions (Figure 7A). No changes in microbleeding or tissue injury were observed in the 0.25 MPa FUS-BBBD and 0.32 MPa FUS-BBBD regions compared with the contralateral hemisphere. Furthermore, cresyl violet staining was performed to evaluate the effect of FUS-BBBD treatment on neuronal cell loss in APP/PS1 mice. No difference in the number of neurons in the CA1, CA3, and DG hippocampal layers was observed between the FUS region and the contralateral region, indicating that sonication parameters used in this study did not affect neuronal cell loss (Figure 7B). These results indicate that FUS-BBBD using two different sonication intensities did not induce vascular or tissue damage in the brain parenchyma and did not increase the number of damaged neurons.

## 4. Discussion

In this study, we investigated the potential synergistic effect of FUS-BBBD-mediated MB delivery to reduce Aβ plaque deposition in APP/PS1 transgenic mice. The role of FUS-BBBD-mediated MB delivery in Aβ plaque clearance and neuroprotection was confirmed. FUS-BBBD-mediated MB delivery effectively reduced Aβ plaque deposition 5 days post-BBBD. In addition, we found that MB delivered via FUS-BBBD induced AQP-4 expression and reduced neural cell damage in the brain parenchyma. A previous study demonstrated FUS-BBBD-induced upregulation of the expression of AQP-4, a water channel protein involved in regulating the water dynamics in the CNS [35]. Given that similar experimental conditions with the conventional BBB were used in the AD disease model, AQP-4 expression increased by approximately 2-fold (*p* < 0.05) in the study by Mun et al. [35] and approximately 1.6-fold (*p* < 0.05) herein, corroborating that FUS-BBBD could alter the localized water dynamics through AQP-4 upregulation in a healthy animal model, as well as in an AD disease model. Overall, the results from this study suggest that enhanced FUS-BBBD-mediated MB delivery decreased the number of Aβ plaques and neural cell damage, potentially via AQP-4 upregulation-mediated activation of the glymphatic system.

Acoustic pressure is one of the major parameters that determine the efficacy and safety of FUS-BBBD that is induced by vibrating microbubbles in the vasculature. In this study, two different acoustic pressures (0.25 and 0.32 MPa) were used to investigate the therapeutic effects of FUS-BBBD-mediated drug delivery based on the safety range of acoustic pressures (0.15–0.46 MPa) established in earlier mouse model studies [45,46]. In 0.32 MPa-treated brain tissue, the contrast-enhanced intensity was 1.55-fold higher than in 0.25 MPa-treated brain tissue (Figure 2). To verify the safety of the selected acoustic pressure, the brain tissue was stained using H&E and cresyl violet dye to confirm tissue and neuronal damage, respectively. Microhemorrhage and neuronal damage were not detected in the 0.25 MPa and 0.32 MPa FUS-BBBD groups. 

In clinical practice, MB administration is recommended at a concentration range of 1–4 mg/kg for ensuring safety and efficacy [10]. According to the study by Riha et al., methylene blue at 4 mg/kg significantly improves long-term behavioral habituation and memory retention after a single administration [47]. Based on a previous report, we selected an administration dose of 4 mg MB per kg body weight for treatment trials of AD mice. Consistent with the previous study [48], MB exhibited weak fluorescence intensity in the brain, indicative of its low BBB permeability. No significant MB concentration-related changes in the fluorescence intensity were observed in the 0.25 MPa FUS group compared with the sham control group (Figure 3A,B). This indicates that the degree of 0.25 MPa-induced BBBD was not sufficient to deliver the drug to the brain parenchymal region. In contrast, the quantity of MB delivered using 0.32 MPa FUS improved 1.43-fold (*p* < 0.0001) compared with the sham control group and 1.32-fold (*p* = 0.0005) compared with the 0.25 MPa FUS group 1 min post-BBBD (Figure 3B). In accordance with a previous study [33], the Aβ plaque deposition decreased by 19.7% in the 0.32 MPa FUS group without MB. Furthermore, MB delivered using 0.32 MPa FUS exhibited an additive effect on plaque reduction efficiency (reaching 40.6% reduction), which is approximately 2-fold higher than the reduction efficiency in the 0.32 MPa FUS group without MB (Figure 4).

Accumulation of Aβ in the brain is the primary cause of AD pathogenesis and is theorized to result from an imbalance between Aβ production and clearance [4]. Growing evidence suggests that glymphatic clearance in the glia-dependent perivascular space can facilitate the removal of brain wastes such as Aβ and tau [37]. Evidence from a series of recent experiments revealed that AQP-4 is responsible for bulk ISF flow in the glymphatic system, and its deletion resulted in Aβ deposition, aggravation of cognitive impairment, and induction of cerebral amyloid angiopathy in APP/PS1 mice [42,43]. The results from this study show that AQP-4 expression was upregulated in both the 0.32 MPa FUS and 0.32 MPa FUS/MB groups 5 days post-BBBD in APP/PS1 mice (Figure 5). The 0.32 MPa FUS/MB combination treatment resulted in a slightly higher AQP-4 signal change (1.7-fold increase in signal intensity) than 0.32 MPa FUS treatment without MB (1.5-fold increase in signal intensity) (Figure 5A,B). Similar to the above results, previous studies on ultrasound treatment demonstrated the role of AQP-4 upregulation in the local regulation of water dynamics in healthy rats [35] and improvement in the glymphatic–lymphatic drainage of Aβ in AD mice [36]. The results from our study suggest that FUS-BBBD could activate the glymphatic pathway via AQP-4 upregulation and induce Aβ plaque clearance for AD treatment. Furthermore, these findings imply that AQP-4 may be a novel therapeutic target for AD, wherein AQP-4 agonists can be used to promote Aβ clearance from the brain interstitium for AD treatment. Although our results indicate an improvement in the pathophysiological symptoms of AD, an important limitation of this study is the lack of behavioral evidence for the effectiveness of FUS-mediated MB delivery. Additional animal behavior tests such as water maze or novel object recognition are required to support the effect of the combination therapy of FUS and MB on specific aspects of memory enhancement.

Progressive Aβ deposition causes dendritic spine loss, promoting massive neuronal loss in the late stages of AD [3]. In the past few years, several studies have reported the neuroprotective effects of MB in preventing structural and functional neural cell damage that is induced by various types of insults [11]. In this study, treatment with MB (4 mg/kg body weight) in combination with 0.32 MPa FUS significantly attenuated neural cell damage in the FUS region compared with the contralateral region 5 days post-BBBD (*p* = 0.037). However, no significant changes were observed upon treatment with only 0.32 MPa FUS (Figure 6). The mechanisms underlying the neuroprotective effects of MB have been established for the mitochondrial metabolic machinery [49]. MB exhibits neuronal protective effects via suppression of mitochondrial dysfunction, oxidative stress, and ATP loss [50]. In addition, MB prevents and improves cognitive impairment via a reduction in Aβ load and mitochondrial dysfunction [12,51]. Furthermore, MB may be engaged in neurogenesis via amelioration of neuroinflammation and promotion of neurite outgrowth and synaptogenesis [52]. In this study, the exact mechanism that ensues post-MB delivery was not established and should be investigated further. However, MB and FUS combination therapy could be a powerful and effective strategy to achieve better therapeutic outcomes in the treatment of AD. 

## 5. Conclusions

In this study, we demonstrated that FUS-BBBD-mediated MB delivery reduced Aβ plaque deposition and neural cell damage in the targeted hippocampal region. We also demonstrated the ability of FUS-BBBD in the absence of a therapeutic drug to reduce the number of Aβ plaques. In contrast, FUS treatment in the absence of MB did not show significant neuroprotective effects in the FUS-targeted hippocampal region. Additionally, this study demonstrated FUS-BBBD-induced AQP-4 upregulation in an AD mouse model and how FUS-BBBD in combination with MB might contribute to increased AQP-4 expression. The results from this study indicate the potential clinical applications of FUS-BBBD in combination with MB for the treatment of AD and provide novel insights that can be used to investigate the mechanisms underlying AD pathogenesis.

## Figures and Tables

**Table 1:** Summary of the experimental groups.

Group	Pressure Amplitude (MPa)	Methylene Blue (4 mg/kg Body Weight; IV)	Sonication (Left Hemisphere/Right Hemisphere)	Number of Animals
1	0.25	MB (−)	Left: FUS (−)	5
			Right: FUS (+)	
2		MB (+)	Left: FUS (−)	6
			Right: FUS (+)	
3	0.32	MB (−)	Left: FUS (−)	6
			Right: FUS (+)	
4		MB (+)	Left: FUS (−)	6
			Right: FUS (+)	

**Table 2:** MR imaging parameters.

3T MRI System (MAGNETOM Skyra; Siemens Healthineers)							
Sequence	Use	TE * (ms)	TR ** (ms)	FOV *** (mm^2^)	Matrix Size	Slice Thickness (mm)	NEX ****
RARE T1-weighted	Detection of BBB disruption	12	500	26 × 45	76 × 128	1.2	32
RARE T2-weighted	Sonication target planning/Detection of edema	96	2500	26 × 45	76 × 128	1.2	32
**9.4T MRI system (BioSpec 94/20 USR; Bruker)**							
**Sequence**	**Use**	TE * (ms)	TR ** (ms)	FOV *** (mm^2^)	**Matrix size**	**Slice Thickness (mm)**	NEX ****
RARE T1-weighted	Detection of BBB disruption	6.5	1500	40 × 40	256 × 256	1.5	3
RARE T2-weighted	Sonication target planning/Detection of edema	96	2500	40 × 40	256 × 256	1.5	2

* TE: echo time; ** TR: repetition time; *** FOV: field of view; **** NEX: number of excitations.
